# Risk stratification and prognostic value of prothrombin time and activated partial thromboplastin time among COVID-19 patients

**DOI:** 10.1371/journal.pone.0272216

**Published:** 2022-08-11

**Authors:** Esayas Tekle, Yemataw Gelaw, Mulat Dagnew, Aschalew Gelaw, Markos Negash, Eyuel Kassa, Segenet Bizuneh, Dessalew Wudineh, Fikir Asrie

**Affiliations:** 1 Department of Medical Laboratory Sciences, Institute of Health Sciences, Wollega University, Nekemte, Ethiopia; 2 Department of Hematology and Immunohematology, College of Medicine and Health Sciences, School of Biomedical and Laboratory Sciences, University of Gondar, Gondar, Ethiopia; 3 Department of Medical Microbiology, College of Medicine and Health Sciences, School of Biomedical and Laboratory Sciences, University of Gondar, Gondar, Ethiopia; 4 Department of Immunology and Molecular Biology, College of Medicine and Health Sciences, School of Biomedical and Laboratory Sciences, University of Gondar, Gondar, Ethiopia; 5 College of Medicine and Health Sciences, University of Gondar Comprehensive Specialized Hospital Laboratory, University of Gondar, Gondar, Ethiopia; 6 College of Medicine and Health Sciences, School of Medicine, University of Gondar, Gondar, Ethiopia; 7 Department of Medical Laboratory Sciences, Institute of Health Sciences, Mizan Tepi University, Mizan Tepi, Ethiopia; Stanford University School of Medicine, UNITED STATES

## Abstract

**Background:**

COVID-19 is a viral disease caused by a new strain of corona virus. Currently, prognosis and risk stratification of COVID-19 patients is done by the disease’s clinical presentation. Therefore, identifying laboratory biomarkers for disease prognosis and risk stratification of COVID-19 patients is critical for prompt treatment. Therefore, the main objective of this study was to assess the risk stratification and prognostic value of basic coagulation parameters and factors associated with disease severity among COVID-19 patients at the Tibebe Ghion Specialized Hospital, COVID-19 treatment center, Northwest Ethiopia.

**Methods:**

A follow-up study was conducted among conveniently recruited COVID-19 patients attended from March to June 2021. Socio-demographic and clinical data were collected using a structured questionnaire and checklist, respectively. Prothrombin time (PT) and activated partial thromboplastin time (APTT) were analyzed by the HUMACLOT DUE PLUS^®^ machine. Descriptive statistics were used to summarize the socio-demographic and clinical characteristics of study participants. Kruskal Wallis tests were used to compare the difference between parametric and non-parametric continuous variables, respectively. The area under the receiver operating characteristic curve (AUC) was used to evaluate the value of PT and APTT in the risk stratification and disease prognosis of COVID-19 patients. Ordinal logistic regression was used to identify the factors associated with disease severity and prognosis. A P-value < 0.05 was defined as statistically significant for all results.

**Result:**

Baseline PT at a cut-off value ≥ 16.25 seconds differentiated severe COVID-19 patients from mild and moderate patients (AUC: 0.89, 95% CI: 0.83–0.95). PT also differentiated mild COVID-19 patients from moderate and severe patients at a cut-off value ≤ 15.35 seconds (AUC: 0.90, 95% CI: 0.84–0.96). Moreover, alcohol drinkers were a 3.52 times more likely chance of having severe disease than non-drinkers (95% CI: 1.41–8.81). A one-year increment in age also increased the odds of disease severity by 6% (95% CI: 3–9%). An increment of ≥ 0.65 seconds from the baseline PT predicted poor prognosis (AUC: 0.93, 0.87–0.99).

**Conclusions and recommendations:**

Prolonged baseline PT was observed in severe COVID-19 patients. Prolonged baseline PT was also predicted to worsen prognosis. An increase from the baseline PT was associated with worsen prognosis. Therefore, PT can be used as a risk stratification and prognostic marker in COVID-19 patients.

## Background

In December 2019, a cluster of severe acquired respiratory syndrome corona virus-2 (SARS-CoV-2) related acute respiratory infections occurred in Wuhan, China [1]. Because of its genomic similarity to the severe acute respiratory syndrome-related coronavirus, the virus has been dubbed SARS-CoV-2, and the disease induced by it has been dubbed coronavirus disease 2019 (COVID-19) by the World Health Organization [2, 3].

Despite a promising prognosis in mildly ill patients, several complications, including acute respiratory distress syndrome, malfunctioning of the coagulation system, and multiple organ failure can lead to death among severely ill patients [4]. Hypercoagulability is a usual pathobiological exhibition in severe COVID-19 patients [5]. Many coagulation profile have been changed in patients with COVID-19 [6, 7].

The coagulation disorder of patients with COVID-19 infection may attribute to a variety of mechanisms. One of the postulated mechanisms is that the virus binds to the angiotensin-converting enzyme-2 (ACE-2) receptor on Type II pneumocytes in the lungs and disrupts the kallikrein system. The binding of the virus to the ACE-2 receptor results in the down-regulation of ACE-2, which results in angiotensin II-mediated vascular dysfunction and a hypercoagulable state in infected people [8]. The other possible mechanism is the virus damage the endothelial and microvascular cells directly, causing inflammation and releasing excessive amounts of cytokines, all of which contribute to the development of a prothrombotic state [9–11]. Autoimmunity may be another potential player that induces antiphospholipid antibody production as verified in various viral infections [12]. Therefore, COVID-19 Patients might show coagulation anomalies which might relate to disease severity and prognosis [13, 14].

The management of critically ill patients has been incredibly hard because of the complexity and the insufficient evidence on the pathogenesis of COVID-19 [15]. Accordingly, laboratory predictors of COVID-19 progression to severe and fatal forms are urgently needed. These predictors will allow for risk stratification of the disease and optimize the allocation of limited human and technical resources in the ongoing pandemic [16, 17]. They also play an important role in the clinical decision, treatment, and management of the COVID-19 outbreak by providing evidence-based data for medical professionals. Studies supported that around 20% to 55% of COVID-19 hospitalized patients had laboratory evidence of coagulopathy and 20% of them had severe coagulation abnormalities [18, 19]. Theresults provided valuable clinical and laboratory features for patients suffering from COVID-19 [20–22].

According to research findings, D-Dimer and fibrinogen levels have been recommended as best coagulation tests in risk stratification and monitoring of COVID-19 patients’ prognosis [23–25]. However, these tests are not readily available in all countries and settings. As a result, identifying the local easily accessible biomarkers such as PT and APTT can be useful to stratify disease severity and prognosis in COVID-19 patients. Moreover, currently in the Ethiopian health system, severe stratification and prognosis of COVID-19 cases are assessed and determined by using the clinical feature of the COVID-19 [26]. Therefore, the main objective of the study was to assess the prognostic and risk stratification value of PT, APTT and factors associated with disease severity among COVID-19 patients.

## Materials and methods

### Study design, setting and population

A follow-up study design was conducted to assess the risk stratification and prognostic value of PT APTT, and factors associated with disease severity among COVID 19 patients at the Tibebe Ghion Specialized Hospital COVID-19 treatment center from March to June 2021. The Hospital is found in Bahir Dar town which is 565 km away from Addis Ababa, the capital city of Ethiopia. The hospital is providing different medical services for more than 5 million people in the region [27]. All real-time reverse transcription polymerization chain reaction (RT-qPCR) confirmed COVID-19 patients at the Tibebe Ghion Specialized Hospital COVID-19 treatment center were the source of populations and the study populations were all RT-qPCR confirmed COVID-19 patients during the study period. But, COVID-19 patients those unable to give biological samples, those who had a predetermined coagulation abnormality and those on anticoagulant drugs therapy before the baseline coagulation determination were excluded from the study.

### Sample size determination and sampling technique

The sample size was determined by using Cohen (1992)’s formula for repeated measurement for ANOVA design [28]. According to this formula, the sample size calculations depend on the effect size, power (1-β), type I error (α), number of groups, number of repeated measurements, and correlation of repeated measurements. Therefore, we use effect size = 0.4, which is considered a large effect size, α = 0.05, power = 0.95, number of groups = 3 (mild, moderate, and severe), number of repeated measurements = 2 (each patient will be observed at baseline and then on the 7^th^ day), and correlation between repeated measurements = 0.5. By inserting these values into the G* Power 3.1.9.2 software, we got a total sample size of 78 (26 in each group). However, since the study was a follow-up study, a 10% loss to follow-up was considered. Therefore, the final sample size required was 86 (a minimum of 29 cases in each group). Accordingly, a sample size of 117 was used for this study (45 milds, 43 moderates, and 29 severe cases). A convenient sampling technique was used to select study participants until the minimum requirement is fulfilled (a minimum of 29 cases in each group).

### Operational definitions

**A coagulopathy.** was defined as any abnormal prolongation and shorting of PT and/APTT. It was determined by using the reference range of PT (10–14 seconds) and APTT (26–36 seconds) at Felege Hiwot Referral Hospital Laboratory. Accordingly, Shorten PT and APTT were difined as less than 10 seconds for PT and less than 26 seconds for APTT wheras, Prolonged PT and APTT were difined as greater than 14 seconds for PT and greater than36 seconds for APTT.

**Alcohol drinker.** Men who consume more than 4 drinks on any day or more than 14 drinks per week or women who consume more than 3 drinks on any day or more than 7 drinks per week [29, 30].

**Mild cases.** mild clinical symptoms and no pneumonia manifestation can be found on imaging [1].

**Moderate cases.** Patients have symptoms like fever and respiratory tract symptoms and pneumonia manifestation can be seen in imaging [1].

**Severe cases.** Include those with any of the following; respiratory distress, respiratory rate ≥30 breaths/min; oxygen saturation ≤ 93% at rest (measured by Pulse Oximetry). Patients with greater than 50% lesion progression within 24 to 48 hours in pulmonary imaging were also treated as severe cases. Moreover, Patients with respiratory failure and mechanical ventilation, shock, and complications from another organ failure that require mechanical ventilation, monitoring and treatment in the ICU were severe cases [1].

**Prognosis.** We did not find a clear definition of the outcome of the illness (Prognosis). Therefore, we use the change of the clinical-stage/severity to define the prognosis. Accordingly, the clinical progression (prognosis) of the patient at day seven was divided into three types: Good prognosis, no change in prognosis, and worse prognosis.

**Good prognosis.** Patients who were severe at admission and progressed to moderate, mild, or cured on the seventh day of hospitalization; those who were moderate at admission and progressed to mild, or cured on the seventh day of hospitalization; and those who were mild at admission and cure on the seventh day of hospitalization.

**No change in prognosis.** Those patients who had the same disease severity/clinical-stage at admission and on the 7^th^ day of hospital admission (no change in disease severity).

**Worse prognosis.** Patients who were mild at admission and progressed to moderate or severe on the 7^th^ day of hospital admission; patients who were moderate at admission and progressed to severe on the 7^th^ day of hospital admission; and patients who were severe at admission and died till the 7^th^ day of hospital admission had a worse prognosis. It also included those patients who had mild, moderate, or severe conditions at baseline and died till the 7^th^ day of hospital admission.

### Data collection procedures

#### Socio-demographic and clinical data collection

A pretested structured questionnaire was used as a tool for the collection of socio-demographic data. Relevant socio-demographic data (gender, age, residence, current marital status, educational status, occupation, alcohol use) and past and current medical history, including the history of chronic disease, were collected by trained nurses. A clinical data and clinical examination of patient’s vital signs like body temperature, blood pressure, pulse rate, respiratory rate, and oxygen saturation percentage were also measured and recorded on a data collection checklist. During follow-up, these vital signs were monitored according to the setup guideline and recorded by nurses on the data collection checklist.

#### Blood sample collection and analysis

*Blood sample collection*. After getting written informed consent from the study participants, 2.7 ml venous blood was collected aseptically from the patients by an experienced medical laboratory technologist from their antecubital vein by using a vacutainer collection system in a 3.2% tri-sodium citrate anti-coagulated test tube. The blood specimen and anticoagulant were mixed gently. Then the platelet-poor plasma, which was used for PT and APTT tests, was obtained by centrifuging at 1500 x g for 15 minutes. The proportion of anticoagulant and blood was 1 part sodium citrate anticoagulant to 9 part blood. To assure quality, the collected blood was checked for proper labeling, hemolysis, and clotting pre-analytically.

*APTT and PT determination*. The APTT and PT were determined at the Felege Hiwot Referral Hospital Laboratory by using the HUMACLOT DUE PLUS^®^ coagulation analyzer (Wiesbaden, Germany). APTT was determined by mixing warmed platelet-poor plasma with warmed APTT reagent and CaCl_2_. The PT reagent, which contains lyophilized thromboplastin of the rabbit brain, was reconstituted by mixing one vial of thromboplastin reagent with one vial of CaCl_2_ buffer solution [30].

### Data quality assurance and control

#### Socio-demographic and clinical data quality assurance

The questionnaire was prepared in English, translated to the Amharic language, and then translated back to English to check for consistency. It was also pre-tested on 5% of COVID-19 patients at Debre-Markos University’s COVID-19 treatment center for its accuracy before actual data collection. The appropriate one-day training was given for all data collectors about the objective of the study, confidentiality issues, study participants’ rights, consenting, and techniques of the interview before data collection. Socio-demographic and clinical data were collected by trained and experienced nurses under the close supervision of investigators. Vital sign measurements (blood pressure, temperature, pulse rate, respiratory rate were measured two times and the mean was taken as the actual measurement. Every collected data was checked for its completeness daily by the investigators.

#### Laboratory examination quality assurance

To maintain the quality of the result, pre-analytical, analytical, and post-analytical precautions of quality and the standard operating procedure (SOP) were strictly followed and quality control results were interpreted according to the rules. All manufacturer instructions and SOP were strictly followed. Every specimen collection procedure was performed aseptically to avoid contamination for both the patient and the specimen, and the entire collected blood sample was checked for appropriate labeling, hemolysis, clotting pre-analytically. Preventive maintenance of the instruments used (Human Cue-due plus analyzer) was done daily, weekly, and monthly by laboratory personnel. All reagents and materials used were checked for their expiration date, leakage, and signs of deterioration (Large flaky particles in the suspension or prolonged prothrombin times on testing normal plasma or controls may indicate product deterioration).

*APTT and PT quality control*. The analytical quality control of the coagulation test was done by running normal (HEMOSTAT CONTROL PLASMA NORMAL) and abnormal quality control (HEMOSTAT CONTROL PLASMA ABNORMAL) material daily before performing tests on patient plasmas. If the results of the controls were not within their reference ranges, patient results were considered invalid and not run/reported.

### Safety management

Data collectors were provided training on COVID-19 prevention, including when to get tested, use of personal protective equipment, and cleaning. Conditions of entry were displayed for any customers or visitors on entry points. Hand sanitizer was provided at multiple locations throughout the workplace. Detergent/ disinfectant surface wipes were provided to clean workstations and equipment used. Bathrooms were stocked with hand soap and paper towels and had posters with instructions on how to wash your hands. There was frequent cleaning of touched areas and surfaces.

Disinfectant solutions were maintained at an appropriate strength and used per the manufacturer’s instructions. Data collectors were wearing gloves when cleaning and washing hands thoroughly with soap and water before and after each activity. In general, the use of the required personal protective equipment like a particulate respirator, mask, gown, gloves, was ensured when contacting the patient and blood sample collection from the patient and also whenever it is required in the process. All intervention procedures were done by sterilized and safe maneuvers.

### Data management and analysis

The data were checked for completeness, cleaned, sorted, and categorized daily before being entered into EpiData version 4.6 and exported to IBM SPSS version 25 for analysis. Socio-demographic and clinical characteristics of study participants were summarized by using descriptive statistics and then presented in tables and text. Categorical variables were given as frequency rates and percentages; continuous variables were expressed as mean and SD (parametric data) or mean rank, median and IQR (non-parametric data). The Shapiro-Wilk test was used to verify the normality of the distribution of continuous variables.

The ANOVA (parametric data) and Kruskal-Wallis H-test (non-parametric data) were used to analyze differences between groups. The post hoc test was used following the ANOVA and Kruskal-Wallis H-test for the parameters that showed a significant difference in the groups to determine which group was exactly the source of the difference (differences between mild, moderate and severe stages of the disease and also between good and worse prognosis groups).

Receiver–operating characteristic (ROC) curve analysis was also performed to determine the area under the curve (AUC) to evaluate the prognostic and stratification values of the PT and APTT. The Youden’s index of the ROC curve was calculated to establish the cut off value of PT and APTT that showed the best combinations of sensitivity, specificity. In the ROC curve analysis, a parameter having the highest AUC was selected as the best prognostic and stratification parameter in COVID-19 patient management. Ordinal logistic regression was used to identify the factors associated with disease severity. In all statistical tests, p-value < 0.05 was considered statistically significant.

### Ethical considerations

The study was conducted after it was reviewed and approved by the Ethical Review Committee of the School of Biomedical and Laboratory Sciences with a reference number *SBMLS/2743/13*. A permission letter to conduct the study was also obtained from Tibebe Ghion Specialized Referral Hospital’s chief executive officer before the commencement of the study, and the study was as per the principles of the declaration of Helsinki II. Written informed consent was also obtained from each study participant and any participant who was not willing to participate in the study was not forced to participate. They were also informed that all the data obtained would be kept confidential by using codes instead of any personal identifiers and used only for the study purpose. Moreover, all laboratory test results were communicated to their physicians for prompt patient management.

## Results

### Socio-demographic and behavioral characteristics

A total of 117 COVID-19 patients were enrolled in this study at admission to the TGSH COVID-19 treatment center. Of the included participants, 77 (65.8%) were male and 94 (80.3%) were urban residents. Education-wise, 49 (41.9%) have attained university or college, while the majority of the participants were government employees, 42 (35.9%), followed by merchants, 27 (23.1%). Of the study participants, 102 (87.2%) were married and 40 (34.2%) had alcohol consumption habits. The patients’ age ranged from 20 to 88 years old with a mean age of 50.62 ±15.39 (**Table 1**).

**Table 1:** Socio-demographic and behavioral characteristics of study participants attending at the Tibebe Ghion Specialized Hospital, COVID-19 treatment center 2021 (n = 117).

Variable	Category	Clinical stage of the disease				P-value^c^
		Mild (n = 45), n (%)	Moderate (n = 43), n (%)	Severe (n = 29), n (%)	Total (N = 117), n (%)	
Age in years	18–35	15 (33.3)	9 (20.93)	1 (3.5)	25 (21.4)	**0.037**
	36–55	17 (37.8)	19 (44.19)	13 (44.8)	49 (41.9)	
	≥56	13 (28.9)	15 (34.88)	15 (51.7)	43 (36.8)	
	Mean (± SD)	44.6 (14.9)	52.5 (14.9)	57.2 (13.8)	50.62 (15.4)	
Gender	Male	26 (57.78)	30 (69.8)	21 (72.4)	77 (65.8)	0.341
	Female	19 (42.2)	13 (30.2)	8 (27.6)	40 (34.2)	
Residence	Urban	34 (75.4)	38 (88.4)	22 (75.9)	94 (80.3)	0.249
	Rural	11 (24.4)	5 (11.6)	7 (24.1)	23 (19.7)	
Educational status	Unable to read and write	2 (4.4)	6 (14.0)	2 (6.9)	10 (8.6)	0.251
	Primary school	11 (24.4)	9 (20.9)	12 (41.4)	32 (27.4)	
	High school	9 (20.0)	10 (23.3)	7 (24.1)	26 (22.2)	
	College and above	23 (51.1)	18 (41.9)	8 (27.6)	49 (41.9)	
Occupation	Farmer	8 (17.8)	5 (11.6)	4 (13.8)	17 (14.5)	0.554
	Housewife	3 (6.7)	3 (7.0)	6 (20.7)	12 (10.3)	
	Merchant	11 (24.4)	9 (20.9)	7 (24.1)	27 (23.1)	
	Government employee	17 (37.8)	16 (37.2)	9 (31.0)	42 (35.9)	
	Others ^b^	6 (13.3)	10 (23.3)	3 (10.4)	19 (16.2)	
Marital status	Unmarried ^a^	9 (20.0)	6 (14.0)	0 (0)	15 (12.8)	.**041**
	Married	36 (80.0)	37 (86.0)	29 (100)	102 (87.2)	
Alcohol use	Yes	9 (20.0)	16 (37.2)	15 (51.7)	40 (34.2)	**.017**
	No	36 (80)	27 (62.8)	14 (48.3)	77 (65.8)	

**c:** Pearson chi-square P-value; **a:** Single, widowed, divorced **b:** Private, student, unemployed

### Clinical characteristics

From the total study population, 52 (44.44%) had co-morbidity. Co-morbidity was significantly higher in moderate and severe COVID-19 patients compared to mild COVID-19 patients (24.4%, 53.5% and 62.1% of mild, moderate and severe patients, respectively) (Chi-Square p-vale = 0.002). The most common co-morbidities were diabetic Mellitus and hypertension (17.1% and 14.5%, respectively). Diabetic Mellitus was significantly associated with disease severity. It was 4.4% in mild COVID-19 patients and 18.6% and 34.5%in moderate and severe COVID-19 patients, respectively (Fishers’ Exact test p-value = 0.002). The most common presenting signs and symptoms were fever (57.26%), headache (59.8%), breathing difficulty (51.3%), cough (65.8%), and myalgia (51.3%). Concerning about the basic coagulation test result, 91 (77.8%) of the patients had prolonged PT and no patient had shortened PT result. The prevalence of prolonged PT was higher in moderate and severe cases (95.3% and 100%, respectively. On the other hand, shorten and prolonged APTT was observed in 54 (46.2%) and 16 (13.7%) patients, respectively. A shortened APTT was more observed in mild cases (77.8%) compare to moderate (32.6%) and severe (17.2%) cases, while prolonged APTT was more observed in severe cases (27.6%) compare to mild (4.4%) and moderate (14.1%) cases. Eight (6.8%) of the patients died within the 7 day follow up peried. The death was significantly higher in severe case (0%, 2.3% and 24.1% in mild, modarete and severe cases, respectively; Fishers’ Exact test p-value <0.001). Seven (6%) patients were lost and their status was not known at day 7 (**Table 2**).

**Table 2:** Clinical characteristics of the study participants attending at the Tibebe Ghion Specialized Hospital, COVID-19 treatment center 2021 (n = 117).

Variable	Category	Clinical stage				
		Mild (n = 45), n (%)	Moderate (n = 43), n (%)	Severe (n = 29), n (%)	Total (117), n (%)	P-value
Co-morbidity^#^	Yes	11 (24.4)	23 (53.5)	18 (62.1)	52 (44.4)	**.002** ^**a**^
	No	34 (75.6)	20 (46.5)	11 (37.9)	65 (55.6)	
Type of co-morbidity						
Hypertension	Yes	3 (6.7)	6 (14.0)	8 (27,6)	17 (14.5)	0.05^b^
	No	42 (93.3)	37 (86.0)	21 (72.4)	100 (85.5)	
Digestive system disorder	Yes	1 (2.2)	4 (9.3)	5 (17.2)	10 (8.5)	0.62^b^
	No	44 (97.8)	39 (90.7)	24 (82.8)	107 (91.5)	
Diabetic mellitus	Yes	2 (4.4)	8 (18.6)	10 (34.5)	20 (17.1)	**0.002**b
	No	43 (95.6)	35 (81.4)	19 (65.5)	97 (82.9)	
Heart disease	Yes	0 (0)	1 (2.3)	2 (6.9)	3 (2.6)	0.183^b^
	No	45 (100)	42 (97.7)	27 (93.1)	114 (97.4)	
Liver disease	Yes	0 (0)	1 (2.3)	0 (0)	1 (0.9)	0.615b
	No	45 (100)	42 (97.7)	29 (100)	116 (99.1)	
Cancer	Yes	0 (0)	1 (2.3)	2 (6.9)	3 (2.6)	0.183^b^
	No	45 (100)	42 (97.7)	27 (93.1)	114 (97.4)	
Tuberculosis	Yes	1 (2.2)	1 (2.3)	2 (6.9)	4 (3.4)	0.554^b^
	No	44 (97.8)	42 (97.7)	27 (93.1)	113 (96.6)	
Renal disease	Yes	0 (0)	1 (2.3)	1 (3.4)	2 (1.7)	0.523^b^
	No	45 (100)	42 (97.7)	28 (96.6)	115 (98.3)	
Epilepsy	Yes	2 (4.4)	0 (0)	1 (3.4)	3 (2.6)	0.464^b^
	No	43 (95.6)	43 (100)	28 (96.6)	114 (97.4)	
Human Immuno virus	Yes	1 (2.2)	3 (7.0)	1 (3.4)	5 (4.3)	0.538^b^
	No	44 (97.8)	40 (93.0)	28 (96.6)	112 (95.7)	
Asthma	Yes	3 (6.7)	5 (11.6)	0 (0)	8 (6.8)	0.146^b^
	No	42 (93.3)	38 (88.4)	29 (100)	109 (93.2)	
Other respiratory disease *	Yes	1 (2.2)	5 (11.6)	4 (13.8)	10 (8.5)	0.134^b^
	No	44 (97.8)	38 (88.4)	25 (86.2)	107 (91.5)	
Presenting signs and symptoms	Fever	9 (20.0)	36 (83.7)	22 (75.9)	67 (57.3)	**<0.001** ^**a**^
	Headache	20 (44.4)	32 (81.4)	18 (62.0)	70 (59.8)	**.016** ^**a**^
	Nausea	6 (13.3)	18 (41.9)	8 (27.6)	32 (27.4)	**.011** ^**a**^
	Breathing difficulty	5 (11.1)	30 (69.8)	25 (86.2)	60 (51.3)	**<0.001** ^**a**^
	Chest pain	6 (13.3)	23 (53.5)	18 (62.1)	47 (40.2)	**<0.001** ^**a**^
	Abdominal pain	12 (26.7)	20 (46.5)	11 (37.9)	43 (36.8)	.153^**a**^
	Cough	28 (62.2)	30 (69.8)	19 (65.5)	77 (65.8)	.757^**a**^
	Sputum production	5 (11.1)	17 (39.5)	12 (41.4)	34 (29.1)	**.003** ^**a**^
	Pharyngalgia	9 (20.0)	23 (53.5)	18 (62.01)	50 (42.7)	**<0.001** ^**a**^
	Myalgia	13 (28.9)	24 (55.8)	23 (79.3)	60 (51.3)	**<0.001** ^**a**^
PT	Normal	24 (53.3)	2 (4.7)	0 (0)	26 (22.2)	**<0.001** ^**b**^
	Shorten	0 (0)	0 (0)	0 (0)	0 (0)	
	Prolonged	21 (46.7)	41 (95.3)	29 (100)	91 (77.8)	
APTT	Normal	8 (17.8)	23 (53.5)	16 (55.2)	47 (40.2)	**<0.001** ^**b**^
	Shorten	35 (77.8)	14 (32.6)	5 (17.2)	54 (46.2)	
	Prolonged	2 (4.4)	6 (14.0)	8 (27.6)	16 (13.7)	
Patient status at day 7	Healed	16 (35.6)	6 (14.0)	1 (3.4)	23 (19.7)	**<0.001** ^**b**^
	On treatment	27 (60.0)	31 (72.1)	21 (72.4)	79 (67.5)	
	Died	0 (0)	1 (2.3)	7 (24.1)	8 (6.8)	
	Lost	2 (4.4)	5 (11.6)	0	7 (6.0)	

**#:** presence of one or more of the disease listed in the type of co-morbidity

**a:** Pearson chi-square P-value

**b:** fisher exact test P-value

*: bronchitis and allergic rhinitis

### Risk stratification values of baseline basic coagulation tests

According to the current study, PT (mean rank: 30.20, 66.09 and 93.17; p-value = 0.001) and APTT (mean rank: 38.03, 66.86 and 79.88; p-value = 0.001), had significant differences between mild, moderate and severe groups, respectively. The Posthoc test of the Kruskal Wallis test also showed that PT was significantly different between mild and moderate, and mild and severe cases. It was lower in the mild case compared to moderate and severe cases (mean rank difference:-35.893 and -62.972; p-value < 0.001, respectively). It was also shown that PT was significantly lower in moderate cases compared to severe cases (mean rank difference: -27.079; p-value = 0.003). APTT was also lower in mild cases compared to moderate and severe cases (mean rank difference; -28.827 and -41.846; p-value < 0.001, respectively). However, APTT had no significant difference between moderate and severe cases (mean rank difference; -13.019; p-value = 0.330) (**Table 3**).

**Table 3:** Comparison of baseline basic coagulation parameters between mild, moderate, and severe COVID-19 patients attending the Tibebe Ghion Specialized Hospital, COVID-19 treatment center 2021 (n = 117).

**Variable**	**Measurement category**	**Clinical stage of the disease**						**P-value**
		**Mild**	**Moderate**	**Severe**		Kruskal-Wallis H		
PT, sec	Median (IQR)	13.8 (13.45–15.1)	16.0 (15.4–17)	18.3 (16.7–20.3)		63.796		< .001***
	Mean rank	30.20	66.09	93.17				
APTT, sec	Median (IQR)	23.7 (22.1–25.25)	28.6 (25–31.5)	29.8 (26.2–36.65)		30.499		< .001***
	Mean rank	38.03	66.86	79.88				
**Posthoc of Kruskal Wallis H test**								
**Parameter**	**severity (I)**	**severity (J)**	**Mean rank difference (I–J)**		**P-value**		**Adjusted P-value**	
PT	Mild	Moderate	-35.893		.000		.000	
		Severe	-62.972		.000		.000	
	Moderate	Severe	-27.079		.001		.003	
APTT	Mild	Moderate	-28.827		.000		.000	
		Severe	-41.846		.000		.000	
	Moderate	Severe	-13.019		.110		.330	

According to the ROC curve analysis, PT at a cut-off point of ≥ 16.25 seconds can differentiate severe COVID-19 patients from mild and moderate COVID-19 patients with a sensitivity of 89.7%, specificity of 76.1%, PPV of 61.7%, and NPV of 100% (AUC: 0.89, 95% CI: 0.83–0.95). PT can also differentiate mild COVID-19 patients from moderate and severe COVID-19 patients at a cut-off point of ≤ 15.35 seconds with a sensitivity of 82.2%, specificity of 86.1%, PPV of 95.75% and NPV of 100% (AUC: 0.90, 95% CI: 0.84–0.96). APTT can differentiate severe COVID-19 patients from mild and moderate COVID-19 patients at a cut-off value of ≥ 25.5 seconds with a sensitivity of 89.7%, specificity of 54.5%, PPV of 43.94% and NPV of 100% (AUC: 0.74, 95% CI: 0.64–0.83). At a cut-off value of ≤ 25.35 seconds, it can also distinguish mild COVID-19 patients from moderate and severe COVID-19 patients, with a sensitivity of 77.8%, specificity of 79.2%, PPV of 90% and NPV of 100% (AUC: 0.79, 95% CI: 0.7–0.88) (**Table 4**).

**Table 4:** The risk stratification values of baseline PT and APTT among COVID-19 patients attending at the Tibebe Ghion Specialized Hospital, COVID-19 treatment center 2021 (n = 117).

Parameter	Category	AUC (95% CI)	Cut-off value	Sensitivity (%)	Specificity (%)	PPV (%)	NPV (%)	Youden Index
PT, Sec	Severe from others	0.89 (0.83–0.95)	≥ 16.25	89.7	76.1	61.7	100	0.658
	Mild from others	0.90 (0.84–0.96)	≤ 15.35	82.2	86.1	95.75	100	0.683
APTT, sec	Severe from others	0.74 (0.64–0.83)	≥ 25.5	89.7	54.5	43.94	100	0.442
	Mild from others	0.79 (0.7–0.88)	≤ 25.35	77.8	79.2	90	100	0.57

### Prognostic value of baseline prothrombin time

Sevene COVID-19 patients were lost during the follow up period and their clinical stage at day 7 was not known. Therefore, the prognostic value baseline PT was asseses only for 110 study participant. It had significant differences between good prognosis, no change in prognosis, and worse prognosis groups (mean rank: 56.21, 44.82, and 74.56; p-value = 0.005, respectively). The Posthoc test of the Kruskal Wallis test also showed that PT was significantly different between patients with worse prognosis and no change in prognosis (mean rank difference: 29.74; p-value = .004). According to the ROC curve analysis, a baseline PT result of ≥ 16.15 seconds could predict a poor prognosis with a sensitivity of 81.3% and specificity of 63% (AUC: 0.72, 95% CI: 0.57–0.87). However, PT had a low predictive value of COVID-19 patients with a good prognosis at a cut-off value of ≤ 16.05 seconds (AUC: 0.47, 95% CI: 0.36–0.58) (**Tables 5** and **6**).

**Table 5:** Comparison of baseline basic coagulation tests between patient prognosis among COVID-19 patients attending at the Tibebe Ghion Specialized Hospital, COVID-19 treatment center 2021 (n = 110).

**Variable**	**Measurement category**	**Prognosis of the patient**						**P-value**
		**Good** (n = 55)		**No change** (n = 39)	**Worse** (n = 16)		Kruskal- Wallis H	
PT, sec	Median (IQR)	15.8 (14.88–17.13)		15 (13.65–16.45)	17 (16.25–20.75)		10.73	**.005**
	Mean rank	56.21		44.82	74.56			
APTT, sec	Median (IQR)	27.2 (24–31.93)		24.9 (22.6–32.6)	29.6 (25.77–32.3)		3.21	.201
	Mean rank	54.99		49.55	65.99			
**Posthoc test of Kruskal Wallis H**								
	**severity (I)**	**severity (J)**	**Mean rank difference (I–J)**			**P-value**	**Adjusted P-value**	
PT	Good	No change	11.39			0.082	0.247	
		Worse	-18.35			0.041	0.124	
	Worse	No change	29.74			**.0.001**	**0.004**	

**Table 6:** Prognostic values of baseline basic coagulation tests among COVID-19 patients attending at the Tibebe Ghion Specialized Hospital, COVID-19 treatment center 2021 (n = 110).

Parameter	Category of prognosis	AUC (95% CI)	Cut-off value	Sensitivity (%)	Specificity (%)	Youden Index
PT, Sec	Worse from others	0.72 (0.57–0.87)	≥ 16.15	81.3	63	0.443
	Good from others	0.47 (0.36–0.58)	≤ 16.05	60	48.3	0.083

### The prognostic value of a gradual change of prothrombin time and activated partial thromboplastin time

**Fig 1:**
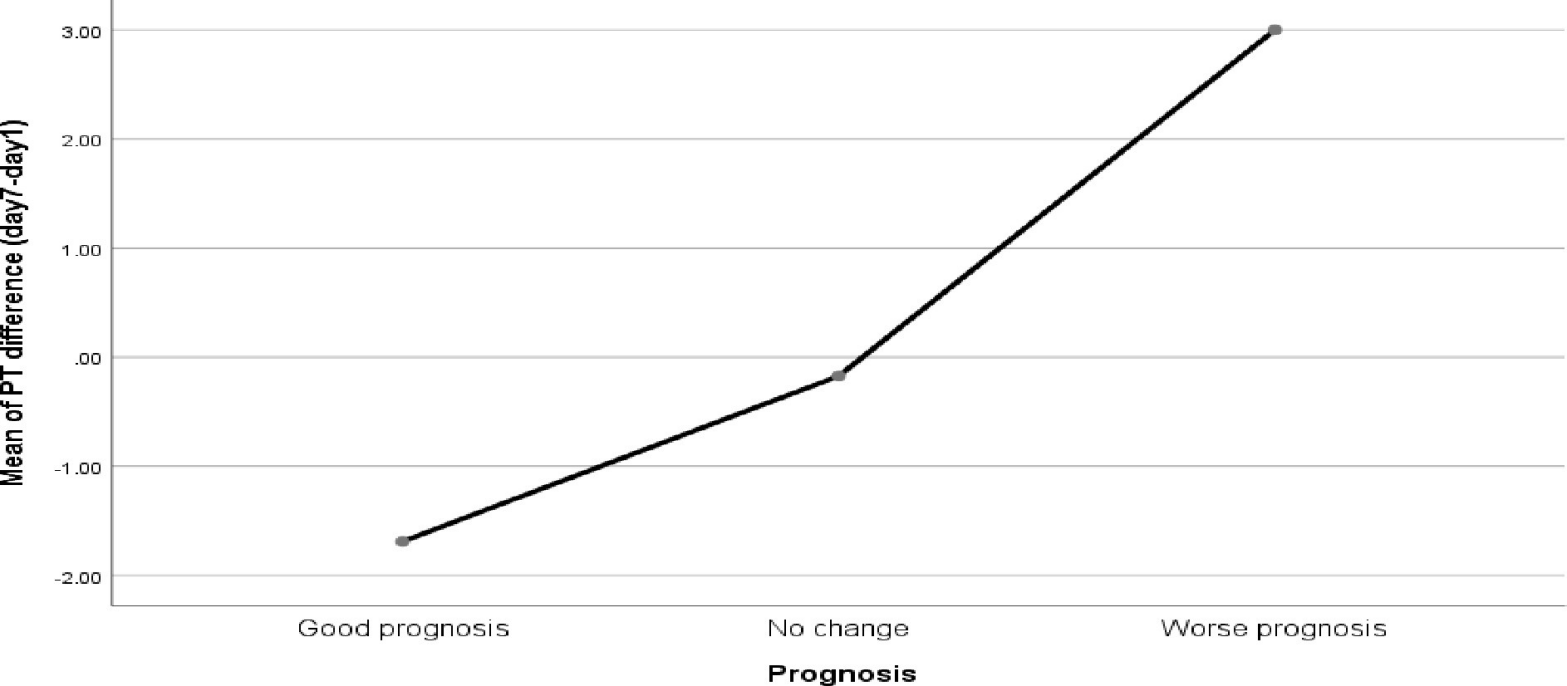
Mean prothrombin time change associated with disease prognosis (7^th^ day—baseline PT).

Seventine COVID-19 patients had no a day 7 PT and APTT due to 7 were lost, 8 were died emeditly after they gave the baseline blood sample and 2 samples rejected due to low volume. Therefore, only 100 COVID-19 patients were had day 7 PT and APTT and the prognostic value of a gradual change of PT and APTTwas assessed using 100 patients. The result showed that patients who had a good prognosis had a low PT result at day 7 compared to baseline PT result. On average, 1.66 (95% CI: -2.23 to -1.09) second was decreased at the 7^th^ day PT compare to baseline PT in patients who had a good prognosis. There was also a slight decrement between the 7^th^ day and baseline PT in patients who had no change in prognosis. But it was statistically insignificant (-0.09 second; 95% CI: -0.79 to 0.61). On the other hand patients with a worse prognosis showed a significant increment in PT result at day 7 compare to the baseline PT; in average, 3.00 (95% CI: 1.24 to 4.76) seconds was increased from the baseline PT). However, the APTT result was not significantly changed with patient prognosis (**Fig 1** and **S1 Table**).

**Fig 2:**
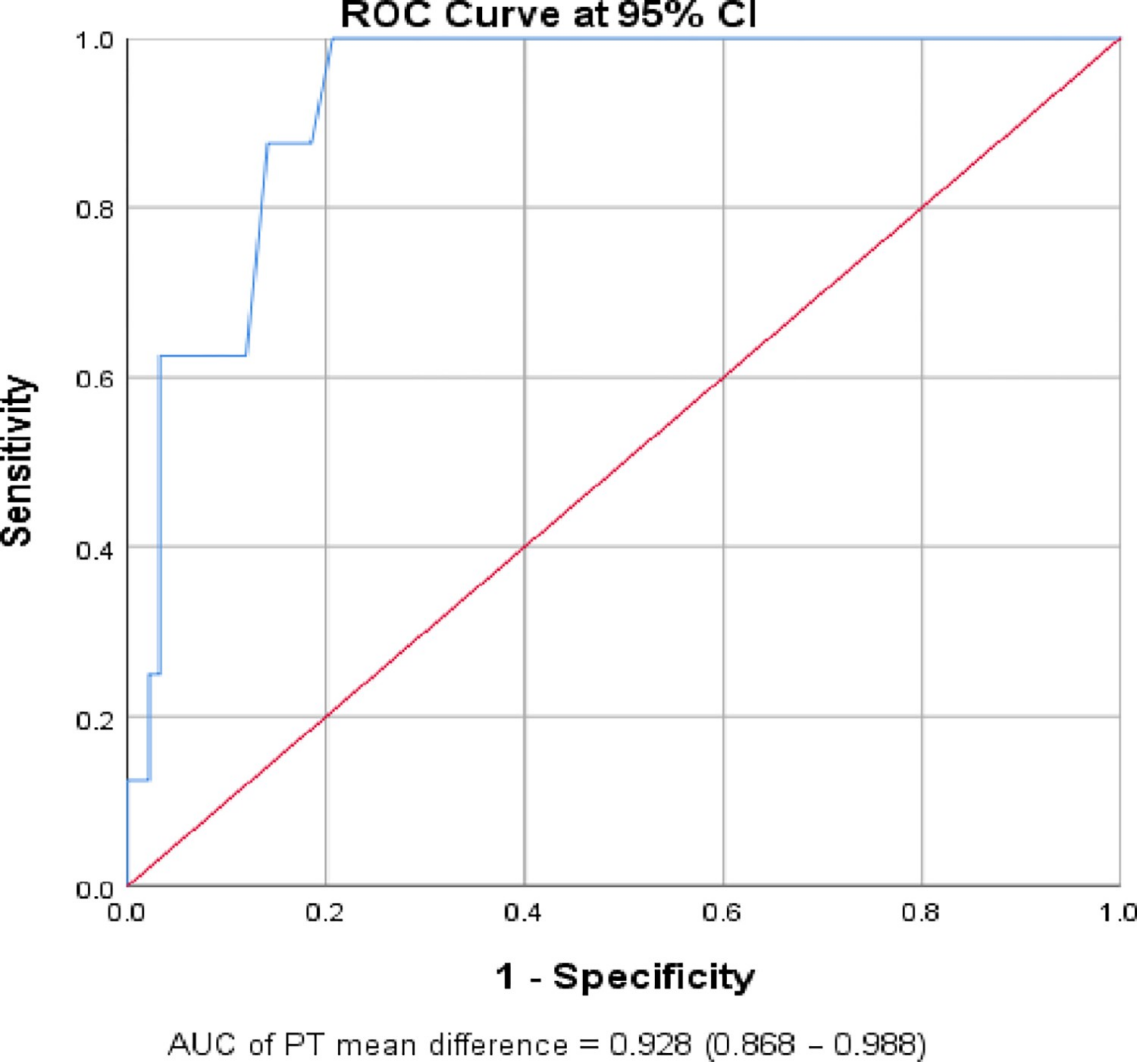
The ROC curve of the gradual change of prothrombin time over the 7th days for prediction of worse prognosis (n = 100).

**Fig 3:**
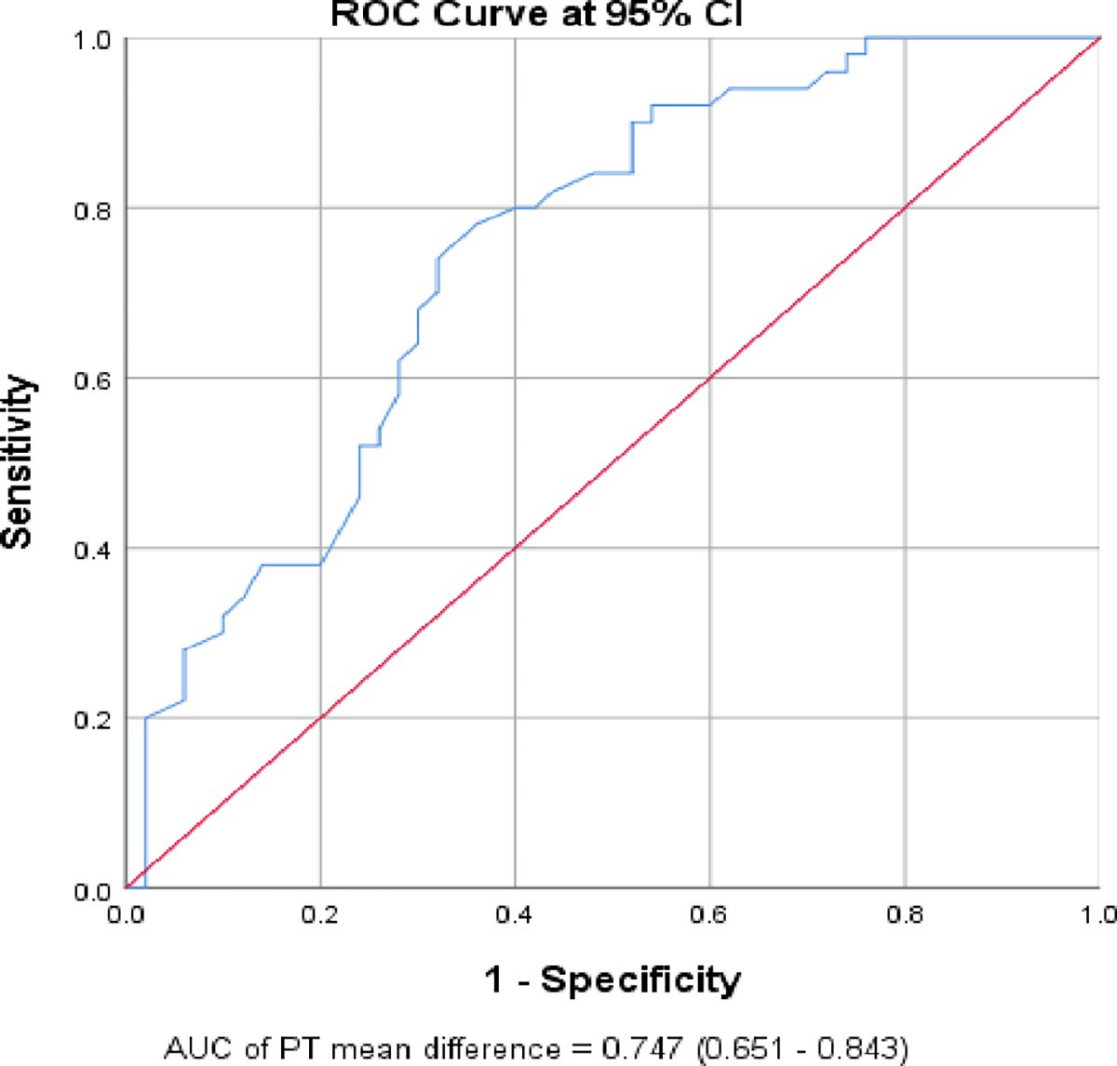
The ROC curve of the gradual change of prothrombin time over the 7th days for the prediction of good prognosis of COVID-19 patients (n = 100).

According to the ROC curve analysis, an increase of ≥ 0.65 seconds from the baseline PT result indicated a worse prognosis with a sensitivity of 100% and a specificity of 79.3% (AUC: 0.93; 95% CI: 0.87–0.99). It also revealed that a mean decrement of ≥ 0.4 seconds from the baseline PT result indicated a good prognosis, with a sensitivity of 78% and specificity of 64% (AUC: 0.75; 95% CI: 0.65–0.84) (**Table 7**, **Figs 2** and **3**).

**Table 7:** The prognostic value of a gradual change of prothrombin time over the 7th-day among COVID-19 patients attending the Tibebe Ghion Specialized Hospital, COVID-19 treatment center 2021 (n = 100).

Parameter	Prognosis category	AUC (95% CI)	Cut-off point	Sensitivity (%)	Specificity (%)	Youden Index
PT mean difference	Worse from others	0.93 (0.87–0.99)	≥ 0.65	100	79.3	0.793
	Good from others	0.75 (0.65–0.84)	≤ -0.4	78	64	0.42

### Factors associated with disease severity and prognosis

In the ordinal logistic regression analysis, COVID-19 disease severity was found to be associated with PT, age and alcohol consumption. A one-second increment in PT increased the odds of COVID-19 severity by 2.47 times more likely (95% CI: 1.81–3.37). The result also showed that one year increment of the patient’s age increased the severity of COVID-19 disease by 6% (95% CI: 3–9%). The odds of COVID-19 disease severity were 3.52 times more likely among alcohol drinkers compared to non-drinkers (95% CI: 1.41–8.81). However, APTT (AOR: 0.99; 95% CI: 0.90–1.08) and co-morbidity (AOR: 1.96; 95% CI: 0.80–4.80) were not significantly associated with disease severity.

On the other hand, the ordinal logistic regression showed that only the PT mean difference was significantly associated with a worse prognosis of COVID-19. A one-second increment from the baseline PT to the 7^th^ day PT increased the odds of a worse prognosis by 1.70 times more likely (95% CI: 1.37–2.12). However, age (AOR: 1.01; 95% CI: 0.99–1.04), alcohol consumption (AOR: 0.50; 95% CI 0.19–1.27), and co-morbidity (AOR: 1.04; 95% CI: 0.43–2.53) were not significantly associated with worsen prognosis of COVID-19 patients (**Table 8**).

**Table 8:** Factors associated with COVID-19 disease severity among COVID-19 patients attending the Tibebe Ghion Specialized Hospital, COVID-19 treatment center 2021.

**Risk factors of disease severity (n = 117)**	**Adjusted Odds ratio (95% CI)**	**P-value**
PT	2.47 (1.81–3.37)	**0.000**
APTT	0.99 (0.90–1.08)	0.798
Age	1.06 (1.03–1.09)	**0.000**
Alcohol use	3.52 (1.41–8.81)	**0.007**
Co-morbidity	1.96 (0.80–4.80)	0.140
**Risk factors of disease prognosis (n = 100)**		
PT change in second	1.70 (1.37–2.12)	**0.000**
Age in year	1.01 (0.99–1.04)	0.300
Alcohol use	0.50 (0.19–1.27)	0.143
Co-morbidity	1.04 (0.43–2.53)	0.930

## Discussion

Thrombotic complications appear to be a major concern among COVID-19 patients [19, 31]. Therefore, in patients confirmed to have COVID-19, useful prognostic information could be obtained by testing basic coagulation parameters including PT, APTT [32].

Various viral infections, such as SARS-Co and influenza virus infection, have been linked to coagulation disorders [33–35]. According to the current study, most (77.8%) of the COVID-19 patients had prolonged PT. The problem was high in moderate and severe COVID-19 patients (95.3% and 100% of the moderate and severe patients, respectively). On the other hand, only 13.7% of COVID-19 patients were showed a prolonged APTT and it was more common in severe COVID-19 patients compared to moderate and mild COVID-19 patients. This finding was supported by the study done in the Islamic Republic of Iran which reported that prolonged PT was common, but not APTT [32]. The finding was also showed that a shortened APTT was found in 46.2% of COVID-19 patients and it was more common in mild cases (77.8% of the mild cases) compare to moderate and severe cases. This might be due to that in the early phase of the disease, more coagulation proteins might be activated and circulated in the peripheral blood and caused a shortened APTT in vitro. However, as the disease advanced these proteins might be consumed and no more shorten APTT might be observed rather prolonged APTT might be observed. In addition, as the severity increases, there might be liver damage which is responsible for the production of most coagulation proteins. A past report showed that 60% of patients developed liver damage due to the SARS epidemic [36]. COVID-19 also belongs to the same coronavirus family which may cause liver injury [37]. The study done in China found that 41.0% and 5.0% of the COVID-19 patients had abnormal liver function tests and liver injury at admission and after 2 weeks of hospitalization, patients with abnormal liver function tests and liver injury increased to 76.3% and 21.5% respectively [38]. The early affected coagulation protein in liver damage is factor VII due to short half-life in the circulation and this causes an early prolonged PT than APTT [39]. This finding was similar to the study findings done in Millennium COVID-19 care and treatment center, Addis Ababa, Ethiopia [40] and Renmin Hospital of Wuhan University [41].

The present study demonstrated that PT at admission had a statistical significance difference between mild, moderate and severe COVID-19 patients (p-value = 0.001). The Posthoc test analysis of the Kruskal Wallis test revealed that PT was significantly lower in mild COVID-19 patients compared to moderate and severe COVID-19 patients (p-value < 0.001). Prothrombin time was also significantly lower in moderate COVID-19 patients compared to severe COVID-19 patients (p-value: 0.003). The ordinal logistic regression analysis demonstrated that a one-second increase in PT increased the odds of the severity of the disease by 2.47 times (95% CI: 1.8, 3.55). The prolongation of PT among patients with the more severe disease might be due to the consumption of coagulation factors. Prothrombin time evaluates the integrity of the extrinsic and common pathways of coagulation and can be affected by the extrinsic and the final common pathway [41–43]. The activation and increment of the coagulation markers have been reported in many viral infections. The increase of the coagulation markers indicates a balance shifted to a procoagulant state. This ultimately leads to the consumption or loss of clotting factors, organ failure and endothelial cell dysfunction [35]. This finding was consistent with previous studies done in China [44–46]. Moreover, other studies demonstrated that prolonged PT was associated with an increased risk of death [24, 45, 47].

On the other hand, other investigations conducted in China [46, 48], and Ireland [49] were contradicted the current findings. They reported that the admission PT had no significant difference between severe and mild COVID-19 patients. The discrepancy could be due to sample size, racial origin, or the usage of anticoagulant medications. There was a sample size and study participant difference between the current study and study done by Guang et al. Study participants in Guang et al. study also took anticoagulant medications before the baseline blood samples were taken, unlike those in our study [46]. However, the discrepancy between the results of this study and those of Huan et al. in China [48] and Helen et al. in Ireland [49] might be due to racial differences in the study populations. Some studies showed that race/ethnicity has major effects on thrombotic risk, with coagulopathy [49–51].

In the ROC curve analysis, baseline PT, at a cut off value of ≥16.25 seconds, had an AUC of 0.89 [95% CI; 0.83–0.95) to stratify severe COVID-19 patients from the mild and moderate cases with a sensitivity of 89.7%, specificity of 76.1%, PPV of 61.7%, and NPV of 100%. It can also differentiate mild COVID-19 patients from moderate and severe COVID-19 patients at a cut-off point ≤ 15.35 seconds with a sensitivity of 82.2%, specificity of 86.1%, PPV of 95.745% and NPV of 100% (AUC: 0.90, 95% CI: 0 0.841–0.959). This finding was supported by the study conducted in China. According to this study, PT (AUC = 0.90) had a good predictive value to stratify mild versus severe/critical patients [46]. Similar multiple studies support that baseline/admission PT had a good risk stratification value among COVID-19 patients [14, 32, 45, 46, 52–55].

The current study also showed that APTT at admission was significantly different between mild, moderate and severe COVID-19 patients (p-value = 0.001). in the Posthoc test analysis, APTT was significantly lower in mild COVID-19 patients compared to moderate and severe COVID-19 patients (p-value < 0.001). However, in the ordinal logistic regression analysis, controlling the confounding factors, APTT was not associated with disease severity (95% CI: 0.88, 1.07). The current finding was consistent with previous research done in China [44, 48]. However, it was inconsistent with other studies conducted in China [45, 46, 48]. The main source of disagreement might be sample size.

The ROC curve in the current study showed that admission APTT cut-off value of ≥ 25.5 seconds had AUC of 0.737 (95% CI; 0.64–0.83) to differentiate severe COVID-19 patients from mild and moderate with a sensitivity of 89.7%, specificity of 54.5%, PPV of 43.94% and NPV of 100%. At a cut off value of ≤ 25.35 seconds, it can also distinguish mild COVID-19 patients from moderate and severe COVID-19 patients, with a sensitivity of 77.8%, specificity of 79.2%, PPV of 90% and NPV of 100% (AUC: 0.79, 95% CI: 0.7–0.88). Likewise, a study conducted in China showed that APTT at (AUC = 0.75) had good stratification ability in mild vs. severe/critical patients [46].

In the ordinal regression analysis, alcohol consumption and patient age were significantly associated with disease severity. The odds of severity were 3.52 times more likely in alcohol drinkers compared to non-drinkers (95% CI: 1.41–8.81). One systematic review and meta-analysis showed that alcohol consumption was significantly increased the risk of acute respiratory distress syndrome [56]. Acute respiratory distress syndrome is a common clinical manifestation of COVID-19 patients [57]. Alcohol can increase the risk of developing acute respiratory distress syndrome through various mechanisms, including alveolar epithelium dysfunction, alcohol-induced oxidative stress and interference of alveolar macrophage function [58]. A study showed that hospitalized patients with pneumonia, having an alcohol-related diagnosis was associated with a greater likelihood of admission and longer length of stay [59]. Chronic alcohol consumption can also induce cilia dysfunction in airways that reduce their ability to clear bacteria and viruses [58].

Additionally, the current study revealed that one year increment in patient age was increased the odds of severity by 6% (95 CI: 3–9%). Recent studies also revealed that the severity of COVID-19 was high in older age groups [31, 55, 60–63]. Aging changes the immune system, mitochondrial function, and hormone levels. The intact function of the immune system and mitochondria is critical to protection against severe COVID-19. Moreover, Co-morbidities associated with old age, and poor nutrition caused old people at additional risk of COVID-19 [64].

The results of the current study also showed that PT was substantially different between patients with a good prognosis, no change in prognosis, and a poor prognosis (p-value = 0.005). It was longer among COVID-19 patients with a worse prognosis compared to those who had no change in prognosis (p-value 0.004). moreover, the ROC curve analysis revealed that a baseline PT result ≥ 16.25 seconds predicted poor prognosis with a sensitivity of 81.3% and a specificity of 63% (AUC: 0.718, 95% CI: 0.571–0.865). This finding was similar to the findings done by Long et al and Jin et al in China [46, 54].

The current study also assessed the value of the gradual change of PT over the 7 days with patient prognosis. Accordingly, PT had a statistically significant gradual change over the 7 days between patients with a good prognosis and those with a worse prognosis. Patients with a good prognosis were showed a gradual decrement in PT (-1.66 second; 95% CI: -2.23 to -1.09), while patients with worsened prognosis had a significantly gradual raised PT result (3.00 seconds; 95% CI: 1.24 to 4.76). On the other hand, patients that did not show a change in their clinical stage/prognosis over the 7 days had no significant change in PT (-0.09 second; 95% CI: -0.79 to 0.61). The ROC curve analysis also revealed that a mean of ≥ 0.65 seconds increment from the baseline PT result predicted a worse prognosis with a sensitivity of 100% and specificity of 79.3% (AUC: 0.93; 95% CI: 0.87–0.99). It also showed that a mean of ≥ 0.4 seconds decrement from the baseline PT result predicted a good prognosis with a sensitivity of 78% and specificity of 64% (AUC: 0.75; 95% CI: 0.65–0.84). In other words, a gradual one-second rise in PT over the first seven days, increased the odds of worse prognosis by 1.65 times (95% CI: 1.33, 2.04). This finding was supported by a study conducted in Chinese patients which showed a gradual rise in PT over the first seven days following admission was associated with patient death, and it remained stable in surviving COVID19 patients [14]. In addition, a study conducted by Danying et al. in China reported that as the severity of the disease increased, PT also increased significantly [45]. However, contrary to the current findings, the study conducted in Finland by Helen et al. found that there was no significant increment in PT in those patients who had a bad prognosis [49]. The discrepancy might be due to the difference in the study population (predominantly Caucasian) and that their patients were already taking low-molecular-weight heparin when they were admitted. Low-molecular-weight heparin is an anticoagulant used in the prevention of blood clots and venous thromboembolism and it shorten the PT by reducing inflammatory reaction [65, 66].

### Strengths and limitations

This study could provide the value coagulation parameters concerning the prognostic and risk stratification among COVID-19 patients. However, the current study had limitations. First, this study was a single-center follow-up study, and the results may not be representative. Second, the sample size was relatively low. Third, we only collected blood coagulation tests at admission and on the 7^th^ day after hospitalization, which may not accurately reflect the continuous dynamic changes in coagulation and prognosis of COVID-19”. A multicenter study with larger sample size is needed to verify the current findings.

## Conclusion

Dynamically monitoring coagulation parameters such as PT and APTT might help in stratifying and predicting severity and COVID-19 patient outcomes. Prolongation of baseline PT can be used as risk stratification and prediction of COVID-19 patients’ outcomes. A gradual rise in PT over the days was also associated with a worse prognosis. Moreover, PT was the better basic coagulation test than APTT to use as risk stratification and prognostic marker. Hence, regularly assessing and screening the coagulation profile of patients with COVID-19 is important for prompt management and minimizing the worse prognosis of COVID-19 patients. COVID-19 disease severity was also associated with older age. We recommend clinicians and policymakers consider the utilization of PT for risk stratification among COVID-19 patients. It is also recommended that more attention be given to patients with older age groups and patients with alcohol consumption habits, as severity is found to be associated with age and alcohol consumption. Furthermore, multicenter studies with large sample sizes are required to verify the prognostic and risk stratification values of basic coagulation parameters among COVID-19 patients.

## Supporting information

S1 Table Comparison of the change of baseline basic coagulation with patient prognosis. (DOCX)

## Abbreviations

APTT: Activated Partial Thromboplastin Time
AUC: Area Under The Curve
CBC: Complete Blood Count
COVID-19: Corona Virus Disease 2019
DIC: Disseminated Intravascular Coagulation
EDTA: Ethylene Diamine Tetraacetic Acid
IQR: Inter Quartile Range
NPV: Negative Predictive Value
PPV: Positive Predictive Value
PT: Prothrombin Time
ROC: Receiver Operating Characteristic
RT-qPCR: Real Time: Reverse Transcriptase-Polymerase Chain Reaction
SARS-CoV-2: Severe Acute Respiratory Syndrome Corona Virus-2
SOP: Standard Operating Procedures
