# Supplementary material for: Risk stratification and prognostic value of prothrombin time and activated partial thromboplastin time among COVID-19 patients
Source: PLoS One. 2022 Aug 11;17(8):e0272216. doi: 10.1371/journal.pone.0272216 (PMC9371343; doi:10.1371/journal.pone.0272216)
Supplement: S1 Table — (DOCX) [file pone.0272216.s001.docx]

| **Variable** | **Study group** | | | | **P-value** |
| --- | --- | --- | --- | --- | --- |
|  | **Good prognosis**, n= 50 | **No prognosis change**, n=42 | **Worse prognosis**, n= 8 | **Kruskal- Wallis H** |  |
| PT mean rank | 38.14 | 57.71 | 89.88 | 26.42 | **0.000** |
| APTT mean rank | 47.48 | 50.56 | 67.19 | 3.088 | 0.214 |

Table 7. Comparison of the change of baseline basic coagulation with patient prognosis (n=100).
